# Interplay of paternal caregiving and screen use habits on early childhood development and children’s tantrums

**DOI:** 10.1186/s13052-024-01802-x

**Published:** 2024-11-05

**Authors:** Emre Sari, Sıddıka Songül Yalçın

**Affiliations:** 1 Department of Family Medicine, Faculty of Medicine, Hacettepe University, Ankara, Turkey; 2 Department of Pediatrics, Faculty of Medicine, Hacettepe University, Ankara, Turkey

**Keywords:** Early childhood development, Child’s media usage, Father’s affect on child

## Abstract

**Background:**

This study aims to examine the association for paternal care and father-child screen use with early childhood development and children’s temper tantrums.

**Method:**

Study file included questions about paternal characteristics, child care, father-child screen habits, and utilized the UNICEF Early Childhood Development Index (ECDI). Factors influencing ECDI-on-track status and children’s responses when screen use was restricted were investigated with Chi-square test and multiple logistic regression.

**Results:**

The study included 464 fathers having children aged 3–4 years. The findings showed that 89.7% of the children were on track in three out of the four ECDI subgroups. When screen use was restricted, 55.6% of the children engaged in another activity, while 44.4% reacted by crying. Multiple logistic regression analysis revealed that the father’s education level, the child’s age and gender, the starting age for screen usage, the child’s reaction to screen restriction, and having three or more books were associated with ECDI. Furthermore, the child’s reaction to screen restriction was related to the child’s and father’s screen time, the presence of three or more books, the adequacy of care, and being on track in the literacy-numeracy ECDI subgroup.

**Conclusion:**

Screen usage habits significantly impact early childhood development and children’s reactions to screen restrictions. These findings underscore the importance of educating fathers about the effects of their own and their child’s media habits, the quality of fatherly caregiving, and the presence of books in fostering positive child development.

**Supplementary Information:**

The online version contains supplementary material available at 10.1186/s13052-024-01802-x.

## Introduction

Parental behavior is highly context-dependent, influenced by geographical conditions, family characteristics, economic status, work-related factors, and societal norms [1–3]. However, the transition from extended to nuclear family structures, increased maternal employment after childbirth and the growing acceptance of egalitarian division of labor have further transformed paternal responsibilities. These changes reflect broader societal shifts in norms and expectations regarding involved fathering [4–8].

Early childhood development (ECD) is shaped by both genetic and environmental factors [9]. However, environmental influences are crucial in helping children reach their potential. Studies have identified several factors that negatively impact ECD, including lack of breastfeeding, limited cognitive stimulation, neglect, stunting, disability, environmental pollution, extreme poverty, suboptimal parenting, marital conflict, and poor caregiver mental health [10–12]. Conversely, positive factors include reduced screen time for children, higher maternal education levels, and better reading habits of mothers [13]. ECD is supported by nurturing care, which ensures a stable environment that meets children’s health and nutritional needs, protects them from harm, and promotes early learning. Nurturing care also includes emotionally supportive and developmentally appropriate interactions [14]. A stable home environment depends on the contributions of the mother, father, and child. Caregiving practices such as feeding, hygiene, cognitive stimulation, responsive parenting, and safety are key aspects of nurturing care. Although mothers often serve as the primary caregivers, fathers also contribute significantly to this process [15].

Opportunities for early learning, as emphasized in the WHO Nurturing Care Framework, are often overlooked, especially in impoverished regions [14]. For example, a 2013 survey in poverty-stricken areas of China found that a significant percentage of young children lacked access to books and toys, which are critical for developmental progress [16, 17]. Father-focused ECD interventions implemented between 2015 and 2019 have shown effectiveness in enhancing parenting knowledge and promoting positive behavioral changes that can benefit children. These behavioral changes include a reduction in physical violence towards children and women, greater use of modern contraception, improved health awareness, higher uptake of prenatal care, and fathers quitting smoking [18–21].

Effective nurturing care interventions that engage all caregivers, including fathers, are crucial for promoting ECD [14, 22]. Across various continents, a study of 69 countries found that, on average, mothers engage in 2.9 out of 6 stimulating activities with their young children, while fathers participate in only 1.3. In every country except Thailand, mothers were more involved in these activities than fathers. Researchers observed that parents in Africa, both mothers and fathers, tend to engage in fewer stimulating activities compared to their counterparts in Europe, Asia, and Latin America [15]. Notably, the percentage of fathers providing high levels of stimulation was lowest in sub-Saharan Africa at 3.9% and highest in Europe and Central Asia at 21%, with an average of 11.9% globally. In comparison, 39.8% of mothers provided high levels of stimulation, with the rates ranging from 14.6% in sub-Saharan Africa to 70.5% in Europe and Central Asia [23].

Fathers were historically seen as having a ‘forgotten contribution to child development [12, 22, 24]. Yet, both parents play key roles in shaping a child’s identity [24]. Research highlights the unique benefits of paternal caregiving on children’s well-being, independent of maternal involvement. Early and increased paternal involvement is linked to emotional, cognitive, and social development, including improved language skills, academic performance, social competence, reduced delinquency and better health outcomes [25–29]. However, in a 2024 study of Syrian refugees in Jordan, father involvement was unrelated to child developmental outcomes [30]. However, other studies in recent years have shown positive effects of father involvement on ECD issues such as emotional, behavioral outcomes, and positive health (immunization, reduction in hyperactivity) [31–34]. This is related to fathers’ parenting behaviors, marital relationships, and psychosocial well-being. Paternal childcare also enhances maternal rest time and mental health, thereby improving parent-child interactions [12, 22, 35].

Psychological theories such as social learning theory and attachment theory explain the relationship between fathers’ involvement and child behavioral outcomes [36, 37]. Social learning theory suggests that children learn by observing and imitating their parents, while attachment theory argues that secure attachment relationships with caregivers, including fathers, lead to improved behavioral outcomes. Paquette’s theory adds that fathers play a unique role in fostering exploration and empowerment in children, known as the father-child activation relationship [38]. In our study, we hypothesize that parental screen time and child screen time are interconnected, as children tend to imitate their parents’ behavior, in line with social learning theory. We also explore whether increased father-child interaction influences ECD according to attachment theory. Our research builds on Paquette’s ideas, among other theories, to examine the specific effects of fathers’ behavior on ECD outcomes. The study’s research questions aim to investigate these theoretical links, particularly how fathers’ care-giving habits and involvement shape children’s development.

Fathers’ screen use habits can also influence childcare practices. On weekdays, children are 3.4 times more likely to exceed the two-hour screen time limit if their father watches TV for at least two hours per day. On weekends, the likelihood increases to 4.8 times [39]. Studies have shown that using screens during meals and fathers using screens as a reward or punishment tend to increase children’s screen time, whereas monitoring screen use and setting clear limits help reduce it [40–42]. It is reasonable to expect that screen use could influence father-child caregiving practices. Fathers’ screen habits may affect how they interact with their children, potentially shaping their engagement, communication, and overall involvement in caregiving. However, no published studies have been identified that specifically evaluate the impact of fathers’ screen use on ECD.

Despite the recognition of the role of fathers in ECD, particularly in low-resource settings [43, 44], there remains a notable gap in understanding how paternal childcare practices interact with father-child screen habits and overall child development. We hypothesize that increased father involvement in caregiving is not only associated with a higher quantity and quality of childcare but also positively influences the development of optimal screen-use habits in children. Specifically, we anticipate that active and engaged paternal caregiving will lead to better development in children, while also fostering healthy screen-time behaviors. Furthermore, we propose that these factors collectively contribute to more appropriate and holistic developmental outcomes in children. This study aims to explore how father involvement in caregiving and screen use habits interact to influence ECD and behavior. The findings are expected to provide valuable insights for developing targeted strategies that promote healthy father-child interactions and optimal child development, ultimately contributing to improved child well-being. These strategies can support the child’s literacy, numeracy, physical development, social-emotional skills, and learning during the preschool period. As a result, ECD interventions can focus on fathers with recommendations for screen use habits included. Planners can consider this data when designing ECD interventions.

## Methods

### Study design and participants

This analytic descriptive study was conducted from January 2023 to April 2023 with fathers of children aged 24–60 months, using an online survey tool. The survey link was distributed through kindergartens and children’s playrooms. To include fathers whose children did not attend kindergarten, participating fathers were encouraged to share the study link within their communities, using a snowball sampling strategy. Fathers were eligible for inclusion if they were the biological father of the child and lived in the same household. Fathers who were not the biological father or did not reside with the child were excluded. Additionally, fathers experiencing extreme poverty, family conflict, or diagnosed mental health problems were excluded, as these factors are known to influence ECD [11, 12]. STROBE guideline was used in the study design.

### Study sample size

Using the G*Power 3.1.9.4 program (Franz Faul, Universitat Kiel, Germany), we determined that a sample size of 400 fathers was needed to achieve a medium effect size (f = 0.25) with an alpha error of 0.05 and a power of 0.95, considering two groups with 20 related factors (covariates). Anticipating a 30% rate of incomplete or incorrect surveys, we planned to reach 520 fathers.

A total of 520 fathers completed the electronic survey. The surveys of 12 single parents, the surveys of 5 children who did not meet the age criteria, and the 39 surveys that were filled out incompletely or incorrectly were excluded from the study. Ultimately, 464 (89.2%) surveys were included in the analysis.

### Ethical considerations

The study protocol was approved by the Local Ethics Committee on 24.01.2023 with the research number GO 22/1314. Informed consent was obtained from all participating fathers. Fathers who accessed the online survey form (Google Forms) and checked the consent box to participate in the study were able to access the survey questions and were included in the study.

### Study tool

The survey form consisted of three main sections: General characteristics of father-child pairs, media usage characteristics, and ECD module.

General Characteristics section included questions about the father’s age, education level, family income according to national wage standards, family type (nuclear or extended), reading habits, total number of children, and details about the child participating in the study such as age, gender, and any physician-diagnosed chronic health problems. Additionally, it queried the amount of time the father spent with the child, whether the father had attended child-rearing courses, and the child’s kindergarten attendance.

The Media Usage Characteristics section collected standardized information on the media habits of father-child pairs [45, 46]. This included whether the child used screens alone or shared screen time with the father, the duration of media use, the age of initial screen exposure, screen use during meals, and its use as a reward or punishment. The father’s knowledge of smart screen usage, how the child imitated sounds and images seen on screens, and the child’s reaction when screen time was restricted were also systematically assessed.

The ECD module is an internationally standardized tool adapted from UNICEF’s Multiple-Indicator Cluster Surveys to assess optimal ECD [47]. It consists of three components: availability of learning materials, provision of adequate care, and the ECD Index (ECDI). As learning materials, the survey queried the number of books and toys available to the child. In our study, children were included in the category of ‘having enough toys’ if 2 out of 3 questions asked for toy availability (1-playing with toys made at home such as dolls or cars, 2-playing with toys bought from stores, 3-playing with household items such as pots and pans or objects found in nature) were answered yes.

The participation in at least 4 learning-promoting activities considered whether the father has engaged in any of the following activities with the child in the past three days: (a) Reading books or looking at picture books together, (b) Telling stories, (c) Singing songs or lullabies, (d) Taking the child outside the home, (e) Playing together, or (f) Naming, counting, or drawing things with the child.

To assess inadequate care, fathers were asked if their child had been left alone with another child under the age of 10 or unattended for more than an hour in the past week. Those who responded ‘yes’ to either question were categorized as providing “inadequate care”.

**Fig. 1:**
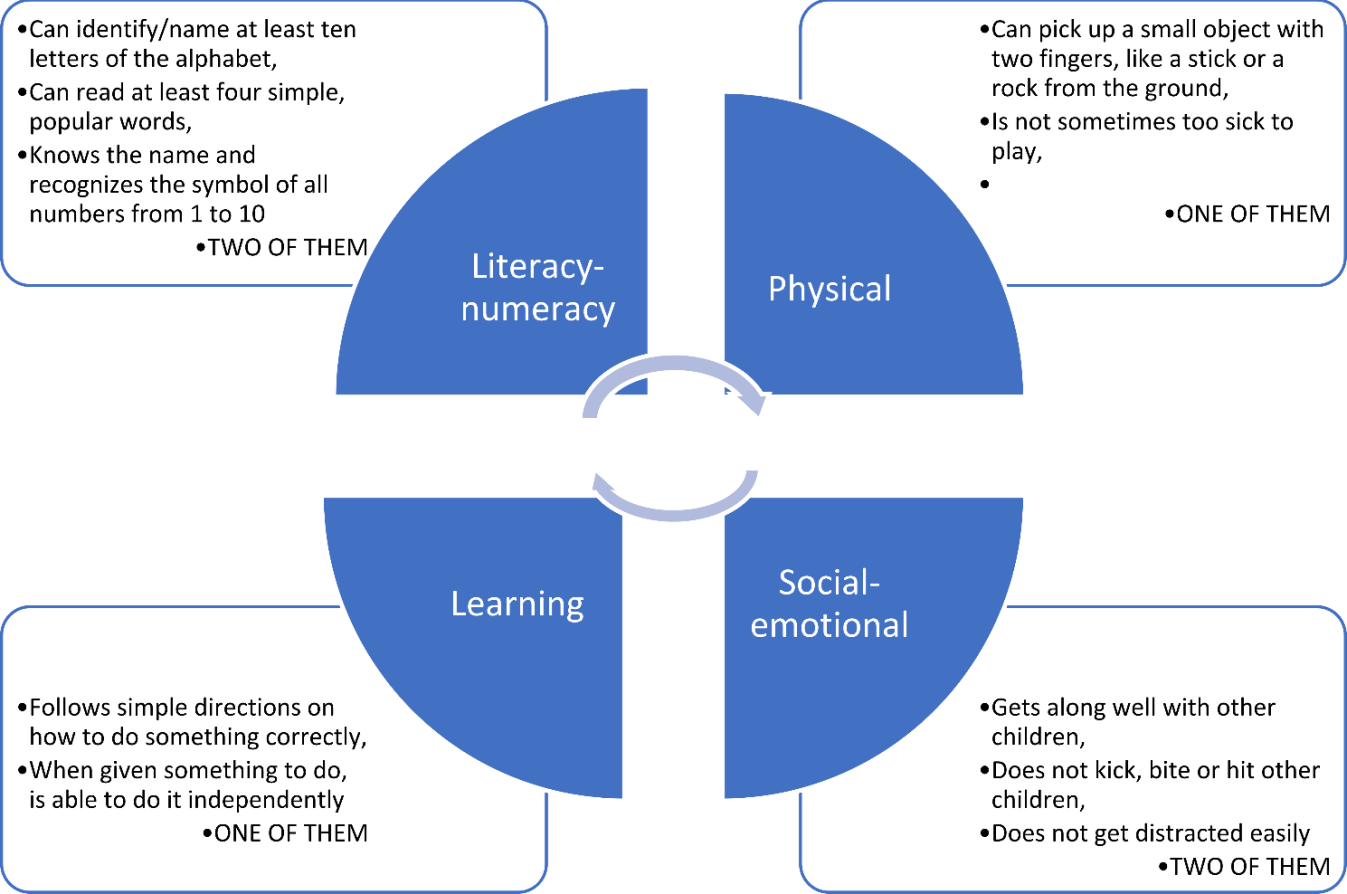
Four domains of early child development index and at least three domains are necessary for the status of “on track”

The ECDI comprises 10 questions that evaluate whether children are meeting developmental milestones across four domains: Literacy-numeracy, Physical development, Social-emotional skills, and Learning. Questions for each domain are outlined in Fig. 1. Children who met the criteria in at least three of these domains were classified as ‘ECDI-on-track’, indicating they were developmentally on track [13, 47].

Prior to the main study, the comprehensibility of the survey questions was tested and refined by administering the preliminary form to 15 fathers. Feedback from this pilot testing was used to adjust the survey forms for clarity and ease of understanding.

### Statistical analysis

Statistical analyses were conducted using IBM-SPSS 23.0 package program (IBM-SPSS Inc., Chicago, IL). Values are given frequencies and percentages. According to father-child variables, ECDI-on-track percentages and child response when not allowed were assessed using the Chi-Square test. When variables with more than two subgroups (3 × 2, 4 × 2 contingency tables) were found to be statistically significant in the Chi-square test, differences between subgroups were assessed using adjusted residuals and Bonferroni correction.

To further explore the factors influencing “ECDI-on-track status”, multiple logistic regression analysis (stepwise method) was conducted. Variables with a chi-square test result of *p* < 0.2 were included in the regression models. These variables included child’s age (4 vs. 3 years), sex (female vs. male), starting age for screen exposure (12–23 months vs. <12 months or unknown vs. ≥24 months), child’s reaction when denied screen use (engaging in another activity vs. crying), screen usage as a reward for the child (yes vs. no), father’s education level (lower school vs. high school vs. university), family income (< NMW vs. ≥NMW, <2x vs. ≥2x NMW), father’s reading habits (no vs. in the past vs. yes), time spent with the child (< 2 h vs. ≥2 h, < 3 h vs. ≥3 h), possession of 3 or more books (yes vs. no), possession of two or more types of toys (yes vs. no), and participation in at least four learning-promoting activities (yes vs. no).

Child-parent variables (independent variables) that had a p-value less than 0.2 in the Chi-Square test for interaction with ‘appropriate reaction when not allowed to use screens’ were selected. We thoroughly assessed collinearity among predictor independent variables to ensure the reliability of the results. Our analysis showed that none of the variables had variance inflation factors exceeding 10, and tolerance values did not approach zero. These findings indicate that there were no significant issues with multicollinearity among the predictors, thereby supporting the validity of our regression models. The selected independent variables were included in a multiple logistic regression analysis using the Stepwise method. These variables included child’s daily screen time (< 1 h vs. ≥1 h, < 2 h vs. ≥2 h, unknown), child’s screen use while eating (no vs. yes), family type (nuclear vs. extended), father’s reading habits (no vs. in the past vs. yes), father’s screen time (< 2 h vs. ≥2 h, < 4 h vs. ≥4 h), screen usage as a reward for the child (yes vs. no), knowledge of smart screen usage signs (yes vs. no vs. partial), possession of 3 or more books (yes vs. no), adequacy of child care (adequate vs. inadequate), participation in at least 4 learning-promoting activities (yes vs. no), and ECDI domains including literacy-numeracy, approaches to learning, and social-emotional development. Odds ratios (OR) and 95% confidence intervals (CI) were calculated for significant findings, with a significance level set at *p* < 0.05.

## Results

### Father, child and care characteristics and ECD

Characteristics of father-child couples were given in Tables 1 and 2. Fathers were between the ages of 25 and 52 years. Of fathers, 71.3% spent 3 h or more with their child on a day when they were not working. 48.5% of the fathers were university graduates. The rate of fathers who knew the meaning of the smart signs they saw on the screen was 74.4% (Table 1).

**Table 1:** The relationship for father characteristics with children’s ECDI-on track-status and doing another activity when not allowed for screen use

			ECDI-on-track		Another activity when not allowed for screen use	
	n	%*	n (%)**	*p*	n (%)**	*p*
	464		416 (89.7)		258 (55.6)	
**Father’s age**,** years**						
< 36	222	47.8	195 (87.8)	0.218	120 (54.1)	0.520
≥ 36	242	52.2	221 (91.3)		138 (57.0)	
**Father’s education level**						
Middle School	92	19.8	78 (84.8)	0.161	48 (52.2)	0.621
High school	147	31.7	136 (92.5)		80 (54.4)	
University	225	48.5	202 (89.8)		130 (57.8)	
**Family income**						
< NMW	68	14.7	59 (86.8)	0.141	36 (52.9)	0.425
≥NMW, <2x	230	49.6	202 (87.8)		123 (53.5)	
≥ 2xNMW	166	35.8	155 (93.4)		99 (59.6)	
**Family type**						
Nuclear	434	93.5	390 (89.9)	0.536	245 (56.5)	0.162
Extended	30	6.4	26 (86.7)		13 (43.3)	
**Number of children**						
1	158	34.0	141 (89.2)	0.705	94 (59.5)	0.310
2	198	42.7	180 (90.9)		110 (55.6)	
≥ 3	108	23.3	95 (88.0)		54 (50.0)	
**Father’s reading habit**						
No	231	49.8	201 (87.0)	0.117	125 (54.1)^a^	0.038
A time ago	185	39.9	169 (91.4)		98 (53.0)^a^	
Yes	48	10.3	46 (95.8)		35 (72.9)^b^	
**Father’s screen time**						
< 2 h/day	76	16.4	68 (89.5)	0.376	51 (67.1)^a^	0.038
≥ 2, < 4 h/day	215	46.3	197 (91.6)		121 (56.3)^ab^	
≥ 4 h/day	173	37.3	151 (87.3)		86 (49.7)^b^	
**Father’s time spent with his child**						
< 2 h/day	67	14.4	53 (79.1)^a^	0.005	33 (49.3)	0.344
≥ 2, < 3 h/day	66	14.2	58 (87.9)^ab^		34 (51.5)	
≥ 3 h/day	331	71.3	305 (92.1)^b^		191 (57.7)	
**Child’s screen use with father**						
Never	25	5.4	21 (84.0)	0.763	18 (72.0)	0.348
Rarely	274	59.1	247 (90.1)		153 (55.8)	
Sometimes	139	30.0	124 (89.2)		73 (52.5)	
Often	26	5.6	24 (92.3)		14 (53.8)	
**Participation in a parenting course**						
Yes	40	8.6	37 (92.5)	0.786	26 (65.0)	0.211
No	424	91.4	379 (89.4)		232 (54.7)	
**Willing to participating in education**						
Yes	194	41.8	171 (91.0)	0.447	107 (56.9)	0.639
No	270	58.2	245 (88.8)		151 (54.7)	
**Screen usage as reward for child**						
Yes	164	35.3	142 (86.6)	0.108	80 (48.8)	0.029
No	300	64.7	274 (91.3)		178 (59.3)	
**Screen usage as punishment for child**						
Yes	177	38.1	158 (89.3)	0.829	81 (45.8)	0.001
No	287	61.9	258 (89.9)		177 (61.7)	
**Knowledge of smart signs**						
Yes	345	74.4	312 (90.4)	0.475	195 (56.5)	0.146
No	33	7.1	30 (90.9)		13 (39.4)	
Some part	86	18.5	74 (86.0)		50 (58.1)	

*row percentage; **column percentage; NMW: National Minimum Wage

^ab^: values having different letter were significantly different in subgroup analysis, *p* < 0.05

**Table 2:** The relationship for child characteristics with children’s ECDI-on-track status and doing another activity when not allowed for screen use

			ECDI-on-track		Another activity when not allowed for screen	
	**n**	%*	n (%)**	*p*	n (%)**	*p*
**Child’s age, years**						
3	130	28.0	110 (84.6)	0.026	72 (55.4)	0.953
4	334	72.0	306 (91.6)		186 (55.7)	
**Gender**						
Male	227	48.9	195 (85.9)	0.009	131 (57.7)	0.372
Female	237	51.1	221 (93.2)		127 (53.6)	
**Kindergarten attendance status**						
Yes	403	86.9	364 (90.3)	0.225	224 (55.6)	0.982
No	61	13.1	52 (85.2)		34 (55.7)	
**Child’s health problem**						
Present	50	10.8	45 (90.0)	0.932	32 (64.0)	0.206
Absent	414	89.2	371 (89.6)		226 (54.6)	
**Age of starting screen use**						
< 12 month/don’t know	194	41.8	169 (87.1)^a^	0.055	101 (52.1)	0.282
12–23 months	60	12.9	51 (85.0)^a^		38 (63.3)	
24 months and beyond	210	45.3	196 (93.3)^b^		119 (56.7)	
**Daily screen time of child**						
< 1 h	43	9.3	42 (97.7)	0.236	33 (76.7)^a^	0.006
≥ 1, < 2 h	137	29.5	124 (90.5)		82 (59.9)^b^	
≥ 2 h	235	50.6	208 (88.5)		116 (49.4)^b^	
No idea	49	10.6	42 (85.7)		27 (55.1)^b^	
**Appropriateness of watched content for child**						
Yes	410	88.4	370 (90.2)^a^	0.014	232 (56.6)	0.503
No	27	5.8	20 (74.1)^b^		13 (48.1)	
No idea	27	5.8	26 (96.3)^a^		13 (48.1)	
**Child’s use of screen alone**						
No idea	21	4.5	17 (81.0)	0.242	8 (38.1)^a^	0.053
Never	98	21.1	93 (94.9)		64 (65.3)^b^	
Rarely	212	45.7	190 (89.6)		120 (56.6)^ab^	
Sometimes	102	22.0	89 (87.3)		53 (52.0)^ab^	
Often	31	6.7	27 (87.1)		13 (41.9)^a^	
**Child’s screen usage while eating**						
Yes	234	50.4	207 (88.5)	0.394	116 (49.6)	0.008
No	230	49.6	209 (90.9)		142 (61.7)	
**Child’s reaction when the screen is banned**						
Doing another activity	258	55.6	242 (93.8)^a^	0.004		
Crying is not allowed	116	25.0	99 (85.3)^b^			
Crying is allowed	90	19.4	75 (83.3)^b^			
**Imitating words and songs he/she hears on media**						
Yes	400	86.2	358 (89.5)	0.784	218 (54.5)	0.232
No	64	16.8	58 (90.6)		40 (62.5)	

*row percentage; **column percentage

^ab^: values having different letter were significantly different in subgroup analysis, *p* < 0.05

Of the children, 86.9% went to kindergartens or playrooms. Starting age for screen usage was younger than 12 months of age or unknown in 41.8% and 50.6% of the children had a screen time 2 h or more. The ratio of fathers who did not know their children’s screen time was in 10.6%. While 88.4% of children watched content suitable for their age, the rate of fathers who did not know whether the content their child watched was appropriate for their age was 5.8%. Of the children, 50.4% were using a screen while eating, 44.4% were crying when they were not allowed to use the screen. The screen was used as a reward in 35.3% of the children and as punishment in 38.1% of children (Table 2).

When the learning materials are examined; while the number of children who owned 3 or more children’s books was 350 (75.4%), and the number of children who owned two or more types of toys was 449 (96.8%).

When evaluated in terms of inadequate care (Table 3); the number of children who were left alone or left with a child under the age of 10 for at least 1 h in the last week was 8.0%.

**Table 3:** The relationship for child-care characteristics, children’s ECDI-on-track status and doing another activity when not allowed for screen use

			ECDI-on-track		Another activity when not allowed for screen	
	**n**	%*	n (%)**	*p*	n (%)**	*p*
Having 3 or more books						
No	114	24.6	93 (81.6)	0.001	51 (44.7)	0.007
Yes	350	75.4	323 (92.3)		207 (59.1)	
Having two or more types of toys						
No	15	3.2	10 (66.7)	0.013	6 (40.0)	0.216
Yes	449	96.8	406 (90.4)		252 (56.1)	
Being left alone for at least 1 h in the last week						
No	439	94.6	396 (90.2)	0.164	251 (57.2)	0.004
Yes	25	5.4	20 (80.0)		7 (28,0)	
Being left with a child under the age of 10 for at least 1 h in the last week						
No	448	96.6	401 (89.5)	1.000	251 (56.0)	0.331
Yes	16	3.4	15 (93.8)		7 (43.8)	
Care of child						
Adequate	427	92.0	385 (90.2)	0.254	244 (57.1)	0.023
Inadequate	37	8.0	31 (83.8)		14 (37.8)	
Participating in at least 4 activities that promote learning						
No	194	41.8	167 (86.1)	0.032	97 (50.0)	0.039
Yes	270	58.2	249 (92.2)		161 (59.6)	
ECDI Literacy-numeracy, on-track						
No	219	47.2			104 (42.5)	0.001
Yes	245	52.8			154 (62.9)	
ECDI Physical, on-track						
No	15	3.2			7 (46.7)	0.479
Yes	449	96.8			251 (55.9)	
ECDI Approaches to learning, on-track						
No	13	2.8			3 (23.1)	0.017
Yes	451	97.2			255 (56.5)	
ECDI Social-emotional, on-track						
No	66	14.2			29 (43.9)	0.039
Yes	398	85.8			229 (57.5)	
ECDI Total, on-track						
No	48	10.3			16 (33.3)	0.001
Yes	416	89.7			242 (58.2)	

*row percentage; **column percentage

In the field of supporting learning, the number of children who participated in at least 4 learning-promoting activities with their fathers was found to be 58.2%.

According to ECDI, the number of children with normal development in at least three of the four developmental domains was found to be 416 (89.7%).

## ECDI-on-track status, child reactions when not allowed and other variables

Tables 1, 2 and 3 shows the relationship between ECDI passes for children aged 3–4 years and children’s response when screen use is not allowed, according to the father, child and care characteristics considered for this study.

It was found that the child’s age, gender, age appropriateness of the content watched, reaction when permission is not given, the father spending time with the child, the child’s possession of learning materials (books, toys), and the father’s activity with the child are associated with the child being ECDI-on-track (*p* = 0.026, *p* = 0.009, *p* = 0.014, *p* = 0.004, *p* = 0.005, *p* = 0.001, *p* = 0.013, *p* = 0.032, respectively; Tables 1, 2 and 3).

Factors associated with the child’s reaction when not allowed for screen use were found to be as child’s screen time, screen use while eating, father’s reading habit, father’s screen time, use of screen as reward and punishment, child’s possession of books, inadequate care, father’s activities with child, total ECDI and literacy-numeracy, approaches to learning, social emotional domains of ECDI ((*p* = 0.006, *p* = 0.008, *p* = 0.038, *p* = 0.038, *p* = 0.029, *p* = 0.001, *p* = 0.007, *p* = 0.023, *p* = 0.039, *p* = 0.001, *p* = 0.001, *p* = 0.017, *p* = 0.039, respectively; Tables 1, 2 and 3).

Multiple logistic regression revealed that the “ECDI-on-track status” interacted with several factors including the father’s education level, the child’s age, gender, age at the start of screen use, appropriate reactions when not allowed screen time, and access to books (Table 4). Children of high school graduate fathers were 2.99 times more likely to be ECDI-on-track (95% CI: 1.17–7.64). Additionally, 4-year-olds had a 2.13 times higher likelihood compared to 3-year-olds (95% CI: 1.09–4.15). Girls were 2.93 times more likely to be ECDI-on-track (95% CI: 1.48–5.81). Children who began using screens before 12 months of age or whose start time was unknown were less likely to be ECDI-on-track than those who started after 24 months [OR(95% CI): 0.29 (0.11–0.76)]. Furthermore, children who engaged in other activities instead of crying when denied screen use were 3.06 times more likely to be ECDI-on-track (95% CI: 1.56–6.04). Lastly, having three or more books increased the likelihood of being ECDI-on-track by 2.56 times compared to having fewer books (95% CI: 1.18–5.55).

**Table 4:** Relationship between child’s ECDI-on-track status and some general characteristics, binary logistic regression analysis, *n* = 464

	AOR	95% CI		p*
		Lower	Upper	
Father’s education level				0.037
High school vs. secondary or lower school	2.99	1.17	7.64	**0.022**
University vs. secondary or lower school	1.07	0.46	2.47	0.884
Child’s age, 4 vs. 3 years	2.13	1.09	4.15	**0.027**
Sex, female vs. male	2.93	1.48	5.81	**0.002**
Starting age for screen				**0.042**
12–23 mo vs. ≥ 24 mo	0.66	0.31	1.43	0.291
< 12 mo or unknown vs. ≥ 24 mo	0.29	0.11	0.76	**0.012**
Child’s reaction when not allowed to use screen, Doing another activity vs. cry	3.06	1.56	6.04	**0.001**
Having ≥ 3 vs. < 3 books	2.56	1.18	5.55	**0.017**
Having ≥ 2 vs. < 2 types of toys	3.29	0.93	11.64	0.065
Constant	0.75			0.704

* Multiple logistic regression (method = stepwise) analysed parameters (*p* < 0.2 in univariate analysis) including factors child’s age (4 vs. 3 years), sex (female vs. male), starting age for screen (12–23 mo vs. < 12 mo or unknown vs. ≥ 24 mo), child’s reaction when not allowed to use screen, (doing another activity vs. cry), screen usage as reward for child (yes vs. no), father education (lower school vs. high school vs. university), family income (< NMW vs. ≥ NMW, <2x vs. ≥ 2NMW, father’s reading habit (no vs. a time ago vs. yes), spending time with his child (< 2 h vs. ≥ 2, <3 h vs. ≥ 3 h), Having 3 or more books (yes vs. no), having two or more types of toys (yes vs. no), participating in at least 4 activities that promote learning (yes vs. no)

When the child-father variables showing an interaction (*p* < 0.2) with “child reactions when not allowed screen use” were included in the analysis, multiple logistic regression identified several associated factors: child’s screen time, screen use during meals, father’s screen time, possession of three or more books, child care, and ECDI literacy-numeracy status (Table 5). Children with less than 1 h of daily screen time were 2.65 times more likely to engage in other activities rather than cry when denied screen use, compared to those with more than 2 h daily (95% CI: 1.22–5.78). Those whose fathers had less than 2 h of daily screen time were 2.01 times more likely to do other activities instead of crying when denied screens, compared to children whose fathers had 4 or more hours of screen time daily (95% CI: 1.11–3.62). Having three or more books increased the likelihood of doing other activities instead of crying by 1.7 times (95% CI: 1.05–2.77). Adequate child care was associated with a 2.15 times higher likelihood of engaging in other activities instead of crying (95% CI: 1.03–4.48). Lastly, children who were ECDI literacy-numeracy-on-track were 1.7 times more likely to engage in other activities rather than cry when denied screen use (95% CI: 1.15–2.52).

**Table 5:** Relationship between “appropriate child reactions when not allowed to use screen” and some general characteristics, binary logistic regression analysis, *n* = 464

	AOR	95% CI		p*
		Lower	Upper	
Child screen time				0.052
Don’t known vs. ≥ 2 h	1.66	0.85	3.26	0.139
< 1 h vs. ≥ 2 h	2.65	1.22	5.78	**0.014**
≥ 1, < 2 h vs. ≥ 2 h	1.32	0.84	2.06	0.229
Child’s screen usage while eating				
No vs. Yes	1.48	1.00	2.18	**0.051**
Father’s reading habit				0.088
A time ago vs. no	0.77	0.51	1.17	0.229
Yes vs. no	1.70	0.83	3.51	0.147
Dad screen time				0.060
< 2 h vs. ≥ 4 h	2.01	1.11	3.62	**0.021**
≥ 2, < 4 h vs. ≥ 4 h	1.36	0.89	2.07	0.158
Having 3 or more books				
Yes vs. no	1.70	1.05	2.77	**0.032**
Child care				
Adequate vs. inadequate	2.15	1.03	4.48	**0.042**
ECDI literacy-numeracy, on-track				
Yes vs. no	1.70	1.15	2.52	**0.008**
Constant	1.97			0.200

* Multiple logistic regression METHOD = BSTEP(COND) analysed parameters (*p* < 0.2 in univariate analysis) child daily screen time (< 1 h vs. ≥ 1, <2 h vs. ≥ 2 h vs. don’t know), child’s screen usage while eating (no vs. yes), family type (nuclear vs. extended), father’s reading habit (no vs. a time ago vs. yes), dad screen time (< 2 h vs. ≥ 2, <4 h vs. ≥ 4 h), screen usage as reward for child (yes vs. no), knowledge of smart signs (yes vs. no vs. some part), having 3 or more books (yes vs. no), care of child (adequate vs. inadequate), participating in at least 4 activities that promote learning (yes vs. no), ECDI literacy-numeracy-on-track (yes vs. no), ECDI approaches to learning-on track (yes ve no), and ECDI social-emotional-on track (yes vs. no)

## Discussion

In our study investigating the relationship between father-child factors, media-usage characteristics, and ECD, we found that 89.7% of children in Ankara, Turkey, were ECDI-on-track. This prevalence is higher than that reported in previous studies in Turkey. The Turkey Demographic and Health Survey (TDHS) 2018 reported an ECDI-on-track prevalence of 74% among children aged 36–59 months [48], while a 2023 study in Afyonkarahisar found 78.1% among mother-child pairs [13]. The higher prevalence observed in our study may reflect the higher education levels of the fathers, an improvement over time or be attributed to advantages such as living in Ankara, the capital city. Differences in ECD outcomes could also be influenced by local factors and the involvement of fathers in learning activities. Another key factor contributing to the high rate of children being ECDI-on track in our study group is their increased access to sufficient books and toys. In our study, 75.4% of children had three or more books, and 96% had at least two toys, which is notably higher than the TDHS figures of 29% and 76% [48], respectively. Furthermore, more children in our study participated in learning activities with their fathers compared to the TDHS (58.2% vs. 16.0%) [48]. This likely contributed to the better ECD outcomes seen in our study. ECD interventions should prioritize access to learning materials such as toys and books. Globally, 43% of children under five in low- and middle-income countries fail to achieve their developmental potential each year, highlighting the local importance of our findings [49].

In our multivariate analysis of father-child pairs, several factors emerged as significant predictors of ECDI-on-track status. Older child age, higher paternal education, female gender, later initiation of screen time, appropriate child reactions when denied screen use, and having more than two books were all associated with higher odds of passing the ECDI. These findings align with previous research emphasizing the importance of parental education, child behaviors, and the home environment in ECD [50]. A previous study of mother-child pairs in Turkey also found that the child’s age, birth order, mother’s education, mother’s reading frequency, and screen time were related to children being ECDI-on-track [13].

In our study, 71.3% of fathers spent 3 h or more with their children daily. This level of paternal involvement reflects a significant engagement in childcare activities. A comparative study in Japan revealed a similar trend, where the time spent by fathers with their children increased gradually over the years, reaching 4.36 h per day in 2021, up from 4 h in previous years [51]. However, despite the initial positive association observed in the univariate analysis between children whose fathers spent 3 h or more with them and higher frequencies of ECDI-on-track status, this significance diminished in further analyses. This suggests that the quality rather than just the quantity of time spent with children may be more crucial for developmental outcomes. Furthermore, our study highlighted that only 58.2% of children engaged in at least four activities promoting learning. This finding underscores the need to enhance opportunities for stimulating interactions and educational activities between fathers and their children. Regarding media usage, our findings revealed that 83.6% of fathers spent two hours or more daily on screens, with one-third spending four hours or more. Additionally, joint screen use between children and fathers constituted at least half of all screen time in 35.6% of cases. It is also known that there is a relationship between parents’ media usage time and their children’s usage time [52]. These results emphasize the pervasive role of digital media in father-child interactions and the potential impact on child development, warranting further investigation into its implications. These findings also underscore the importance of encouraging meaningful interactions and educational activities between fathers and children, while also highlighting the risks posed by intense screen time in the context of modern parenting. Future studies should continue to explore these dynamics to identify strategies that optimize developmental outcomes for children.

In our study, more than half of the children were found to have daily screen times of 2 h or more. Approximately one-fifth of the children watched screens with one parent, while 28.7% spent at least half of their screen time alone. We did not find a significant relationship between screen time duration and parental accompaniment with being on track for the ECDI. However, it’s possible that fathers’ screen time could negatively impact the quality of time spent with their children. Father’s excessive screen use is reported to be associated with lower scores on the preschool language scale for both receptive and expressive language [34]. Recently, a mother-child pair study demonstrated that children who used screens for 2 h or less were 2.04 times more likely to be on track for the ECDI [13]. Additionally, our study reported that 5.8% of children engaged in inappropriate media use, and in univariate analyses, these children had significantly lower rates of being ECDI-on-track. Moreover, among 3-4-year-old children with prolonged and solitary screen time, exposure to educational videos might explain why their ECDI outcomes were not adversely affected.

Furthermore, we found that children whose fathers spent 3 h or more with them had higher rates of being ECDI-on-track compared to those whose fathers spent less than 2 h. Conversely, children whose fathers provided inadequate care, including leaving them alone, were less likely to be ECDI-on-track, even after adjusting for confounding factors. This underscores the positive impact of fathers’ involvement in childcare and spending quality time with their children on ECD, aligning with findings from several other studies [29, 43, 53]These results highlight the complex interplay between screen time, parental involvement, and developmental outcomes in children. While the content of the programs (e.g., educational vs. entertainment) children watch may also play a role in their development, our study focused solely on screen time duration, limiting our ability to comment on this aspect. The importance of promoting balanced screen use and active parental involvement is emerging as important factors in supporting healthy ECD. Moreover, despite only 7.1% of fathers reporting a lack of knowledge about smart signs, only a fifth of them allowed their children to use screens alone, and 5.8% were unaware of what their children were watching. This highlights a knowledge gap among fathers regarding the impact of media usage on child health.

It is well-established that stimuli such as books and toys play a crucial role in ECD [14, 16, 17]. In our study, we observed that a significant proportion of children lacked adequate cognitive stimulation. Specifically, 24.6% of children had fewer than 2 books, and 3.2% did not have two or more types of toys. This finding suggests limited access to cognitive enrichment resources among the children in our sample. Our analysis revealed that children who had access to toys and books were significantly more likely to pass the ECDI in univariate analysis. However, when considering multiple factors simultaneously, having 3 or more books emerged as the most influential predictor of ECDI success, maintaining its significance even after adjusting for potential confounders. Comparatively, a study focusing on mother-child pairs reported that 75.0% of children did not have access to books, and 10.4% lacked toys, highlighting these deficits as risk factors for developmental delays [17]. Allel et al. examined data from 68 low- and middle-income countries (LMICs) using multivariate regression analysis to explore how various ecological, socioeconomic, and health-related factors are linked to the ECDI [54]. The research revealed that factors like attending early childhood education, having books at home, and more equitable income distribution positively influence development. The study highlights the importance of socioeconomic conditions and nurturing care in shaping developmental outcomes for children aged 3–5 [54]. Limited access to books and toys in the absence of interaction with the father has been consistently associated with delayed development, especially in children under 3 years of age. These findings underscore the critical importance of ensuring children have adequate access to stimulating materials such as books and toys early in life [16]. On the other hand, the father’s reading belief is related to the child’s media usage habits [45]. Addressing these gaps can potentially mitigate developmental delays and promote healthier cognitive development in young children. Future research and interventions should prioritize improving access to cognitive stimulation resources, especially for vulnerable populations, to support optimal ECD outcomes.

The richest households had less father stimulation for their children than the poorest households [23]. The literature suggests that fathers who are more educated and economically advantaged tend to be more involved in childcare [55, 56]. In our study, we observed a non-significant increase in the ECDI pass rate as income levels rose. We excluded fathers experiencing extreme poverty, domestic conflict, single parenthood, or diagnosed mental health issues from our sample. These exclusions were based on previous findings indicating that conditions such as extreme poverty, suboptimal parenting, marital conflict, and poor caregiver mental health can significantly hinder healthy ECD [10, 11]. These factors were carefully considered to ensure that our study focused on a more homogeneous group, minimizing potential confounding variables that could obscure the relationship between income and ECD outcomes. Therefore, access to learning materials, which may be related to income status and likely to affect ECD results, may have been found to be higher in our study. At the same time, father participation may have been higher because extremely poor fathers were not included in the study. While we did not find a statistically significant association between income and being ECDI-on-track percentages in our study involving educated fathers, the impact of income on child development remains a critical area for further investigation and intervention.

In our study, we did not find a significant relationship between the number of children and being ECDI-on-track. This may be attributed to the fact that the families included in our study typically had between 1 and 4 children, thus limiting the variability needed to detect such a relationship.

When screen was banned and after being told they can’t use screens, 54.4% of children were found to throw a tantrum, crying and protesting loudly. We observed a positive relationship between several factors and appropriate child behavior in relation to screen time management. Specifically, children who had less than 1 h of daily screen time, did not use screens while eating, had fathers with less than 2 h of screen time, possessed three or more books, received adequate childcare, and scored on track in ECDI literacy-numeracy were more likely to engage alternative activities when not allowed to use screens. This suggests that these associated factors contribute positively to children’s behavioral development by reducing excessive screen use. In addition, increased risks for child’s tantrums over screen time restrictions tantrum were associated with using screens as rewards or punishments and children predominantly using screens alone. Interestingly, these associations were not significant when covariants included, possibly indicating that these cases engage in more prolonged screen time and uncontrolled screen usage behaviors of fathers. Research on how parents manage their children’s screen time is limited [30, 46, 57–59]. Halpin et al. study has examined children’s behavior when screen time was banned, finding that dysfunctional parenting styles, such as laxity and overreactivity, were associated with increased behavioral problems related to screen use [30]. Griffith et al. demonstrated that integrating screen media interventions with adapted parenting programs can effectively manage screen use in young children with externalizing behavior problems in a priliminary study [58]. Similarly, a scoping review (*n* = 16 studies) found that parents who reported higher self-efficacy in task-specific areas related to screen time tended to have children with less screen time and were more likely to implement mediation strategies in accordance with public health guidelines [59]. Further research focusing specifically on fathers could provide deeper insights into their role in managing screen use and its impact on ECD. This observation highlights the need for further exploration into how different screen time management strategies impact children’s behavior and whether effective methods can mitigate these negative responses. Comparing these findings with behavioral management practices in other settings could provide valuable additional insights.

### Strengths and limitations

As a strenghts, the examination of individual characteristics such as paternal screen usage, participation in fatherhood or child-rearing courses, and paternal child caregiving provides valuable insights into their impact on child development. The study uniquely investigates the child’s reaction, tantrums, when denied screen use alongside paternal factors, which contributes novel perspectives to understanding behavioral development. However, a notable limitation is the high level of education among the majority of fathers in our sample. This demographic skew may limit the generalizability of our findings to the broader population, particularly those with lower educational attainment. Data were primarily gathered through self-reporting by fathers, which introduces subjectivity. The level of paternal involvement was assessed solely through paternal reports, which could be influenced by paternal perspectives and memory biases. Research in refugee and low-resource communities in Lebanon has shown discrepancies between maternal and paternal perceptions of paternal involvement in childcare; fathers tend to rate themselves as 13% more involved than reported by mothers [51].

On the other hand, the shift from a significant univariate association between paternal involvement (spent time) and ECDI-on-track status to a non-significant result in multivariate analyses suggests that the quality of paternal involvement, rather than just its presence, may be a key factor. We assessed father involvement as time spent with the child, which is consistent with our current study [43]. However, we did not assess the quality of engagement in detail. Further exploration of what constitutes high-quality paternal involvement and how it could be measured would provide a more nuanced understanding. Incorporating qualitative data or case studies in future research could offer deeper insights into the effectiveness of paternal interactions. Additionally, there might be unmeasured confounding variables, previously unforeseen factors, and biases in the self-reported data which represent limitations and these could impact the results. These factors underscore the importance of interpreting our findings carefully and highlight the need for future studies to employ more objective measures and include diverse socioeconomic backgrounds to enhance the robustness and applicability of the results.

## Conclusion

As a conclusion, this study has revealed significant findings regarding the association between fathers’ childcare practices on child development and child tantrums. In our study, we found that 4-year-old children, female children, those whose fathers have higher education levels, who start using screens later, engage in alternative activities when screen use is restricted, and have three or more books, exhibit better developmental outcomes. Furthermore, both the child’s and father’s screen time, having three or more books, inadequate caregiving, and ECDI literacy-numeracy scores were associated with how children react when not allowed to use screens. These findings underscore the importance of educating fathers about the impact of their own and their child’s media habits, the quality of fatherly caregiving, and the presence of books in fostering positive child development. It is crucial to enhance the quality and frequency of fatherhood training provided by public and non-governmental organizations, aiming to involve more fathers in these programs. In these trainings, fathers can be informed about children’s ideal screen use and screen management helping translate the study’s findings into real-world applications and supporting positive changes in practices related to screen time and paternal involvement. Additionally, healthcare providers should routinely inquire about the media usage habits of children and parents, offering guidance on optimal media practices for enhancing child well-being.

## Electronic supplementary material

Below is the link to the electronic supplementary material.

Supplementary Material 1

## Data Availability

The datasets used and/or analysed during the current study are available from the corresponding author on reasonable request. For access to the files, please send an e-mail request to siyalcin@hacettepe.edu.tr.
