# Supplementary material for: Interplay of paternal caregiving and screen use habits on early childhood development and children’s tantrums
Source: Ital J Pediatr. 2024 Nov 5;50:230. doi: 10.1186/s13052-024-01802-x (PMC11536929; doi:10.1186/s13052-024-01802-x)
Supplement: Supplementary file 1 — Supplementary Material 1 [file 13052_2024_1802_MOESM1_ESM.docx]

ITJP-D-24-00573
Interplay of Paternal Caregiving and Screen Use Habits on Early Childhood Development and Children's Tantrums
Emre Sarı; Siddika Songül YALÇIN
Italian Journal of Pediatrics

Dear editor

Thank you for yoour help, corecions were below,

Corrected manuscript was submitted again.

We did not understand where to upload the 3-line letter to the editor. We also uploaded it as a personal cover.

Best Regards

Dr. Songül YALÇIN

C1. Under the "Ethics approval and consent to participate", please provide the missing statement below.- A statement to confirm that all methods were performed in accordance with the ethical standards as laid down in the Declaration of Helsinki and its later amendments or comparable ethical standards.

R1. We corrected as recomended. "Ethical approval and consent to participate: All methods were performed in accordance with the ethical standards as laid down in the Declaration of Helsinki and its later amendments or comparable ethical standards. This study protocol was examined by Hacettepe University Medical Faculty Non-Interventional Clinical Research Ethics Committee and was approved (GO 22/1314). Fathers who participated in the study signed online voluntary consent form."

C2. Missing Figure Citation     The position of Figure Citation (Fig. 1) is not defined in the manuscript.

R2. Figure 2 should be Figure 1 in the manuscript. It is corrected.

C3. Missing Figure 2. We notice that Figure 2 has been referenced in the main text of your manuscript file but this figure has not been included with your submission. Please either upload this figure as a separate 'Figure' file and include a corresponding legend in the main manuscript file or update the references in the main text of your manuscript accordingly. The figure file should be in editable file format, this file size is adequate for extremely high-quality figures.

R3. Figure 2 is not present in the manuscript; it was mistakenly referenced in the text. It has been corrected to Figure 1 in the text.
